# Author Correction: Unraveling the epigenetic code: human kidney DNA methylation and chromatin dynamics in renal disease development

**DOI:** 10.1038/s41467-024-46661-6

**Published:** 2024-03-21

**Authors:** Yu Yan, Hongbo Liu, Amin Abedini, Xin Sheng, Matthew Palmer, Hongzhe Li, Katalin Susztak

**Affiliations:** 1 Renal, Electrolyte, and Hypertension Division, Department of Medicine, University of Pennsylvania, Perelman School of Medicine, Philadelphia, PA 19014 USA; 2 Institute for Diabetes, Obesity, and Metabolism, University of Pennsylvania, Perelman School of Medicine, Philadelphia, PA 19014 USA; 3 Department of Genetics, University of Pennsylvania, Perelman School of Medicine, Philadelphia, PA 19014 USA; 4 Kidney Innovation Center, University of Pennsylvania, Perelman School of Medicine, Philadelphia, PA 19014 USA; 5 Department of Epidemiology and Biostatistics, Perelman School of Medicine, Philadelphia, PA 19014 USA; 6 Department of Pathology, Perelman School of Medicine, Philadelphia, PA 19014 USA

**Keywords:** Kidney diseases, Medical research

Correction to: *Nature Communications* 10.1038/s41467-024-45295-y, published online 29 January 2024

The original version of this article omitted an accession from the data availability statement. The following has been added to the data availability statement in the corrected version: “The human kidney single cell ATAC-seq data are available in GEO under accession number GSE211785.” This has been corrected in both the PDF and HTML versions of the Article.

