# Supplementary material for: Unraveling the epigenetic code: human kidney DNA methylation and chromatin dynamics in renal disease development
Source: Nat Commun. 2024 Jan 29;15:873. doi: 10.1038/s41467-024-45295-y (PMC10824731; doi:10.1038/s41467-024-45295-y)

Supplementary Information

Unraveling the Epigenetic Code: Human Kidney DNA Methylation and Chromatin Dynamics in Renal Disease Development

Yu Yan, Hongbo Liu, Amin Abedini, Xin Sheng, Matthew Palmer, Hongzhe Li, and Katalin Susztak

Supplementary Fig. 1. Association of fibrosis and eGFR and histology parameters.
a. Correlation of percent fibrosis (as % in y-axis) and mean eGFR (ml/min/1.72m2) of the analyzed samples

b. Hierarchical clustering of clinical and histological variables. Note the relationship between different variables.

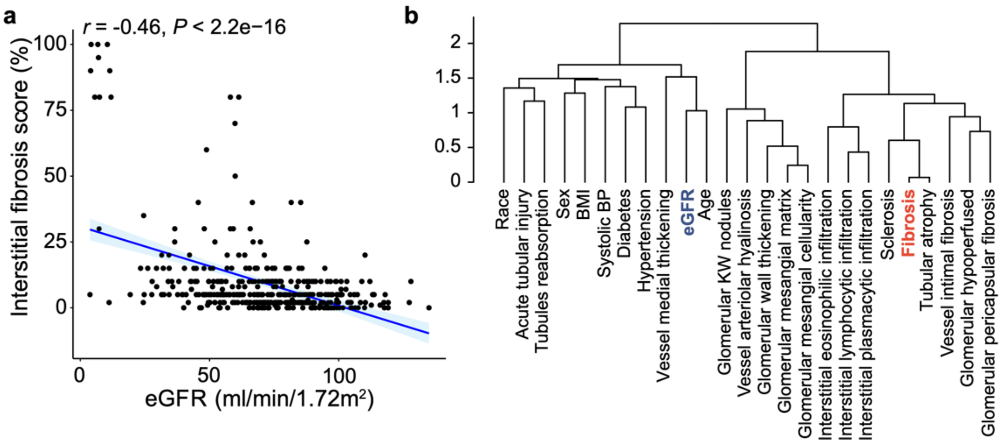

Supplementary Fig. 2. Localization and characteristics of the most significant fibrosis EWAS signal at cg18566594.
Top panel shows the chromosomal location and CCND2-AS1 gene followed by human kidney histone modification tracks (H3K4me3 and H3K27ac) followed by ChromHMM track.

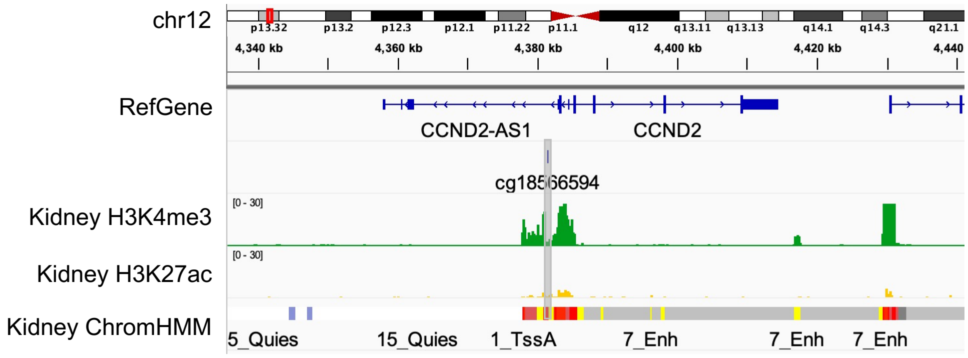

Supplementary Fig. 3. Epigenome-wide association analysis (EWAS) identified methylation changes associated with kidney function (eGFR).

a. Manhattan plot of eGFR EWAS in 399 human kidney samples. The x-axis represents the chromosomal location of the CpG probes and the y-axis is the -log10(*P* value) of eGFR and methylation association. The epigenome-wide significance level ($P < 9.42 \times 10^{-8}$) is indicated by the red line and significant CpGs are highlighted as rectangles.

b. Volcano plot showing the association between eGFR and methylation changes. The x-axis represents the effect size (from the linear regression) of each CpG probe with eGFR and the y-axis indicates the strength of the association (-log10(*P* value)). Each dot corresponds to one probe, with red dots representing hypermethylated probes and cyan dots representing hypomethylated probes that are associated with higher eGFR.

c. Localization and characteristics of the most significant eGFR EWAS signal at cg27630540. Top panel shows the chromosomal location and CCND2-AS1 gene followed by human kidney histone modification tracks (H3K4me3 and H3K27ac) followed by ChromHMM track.

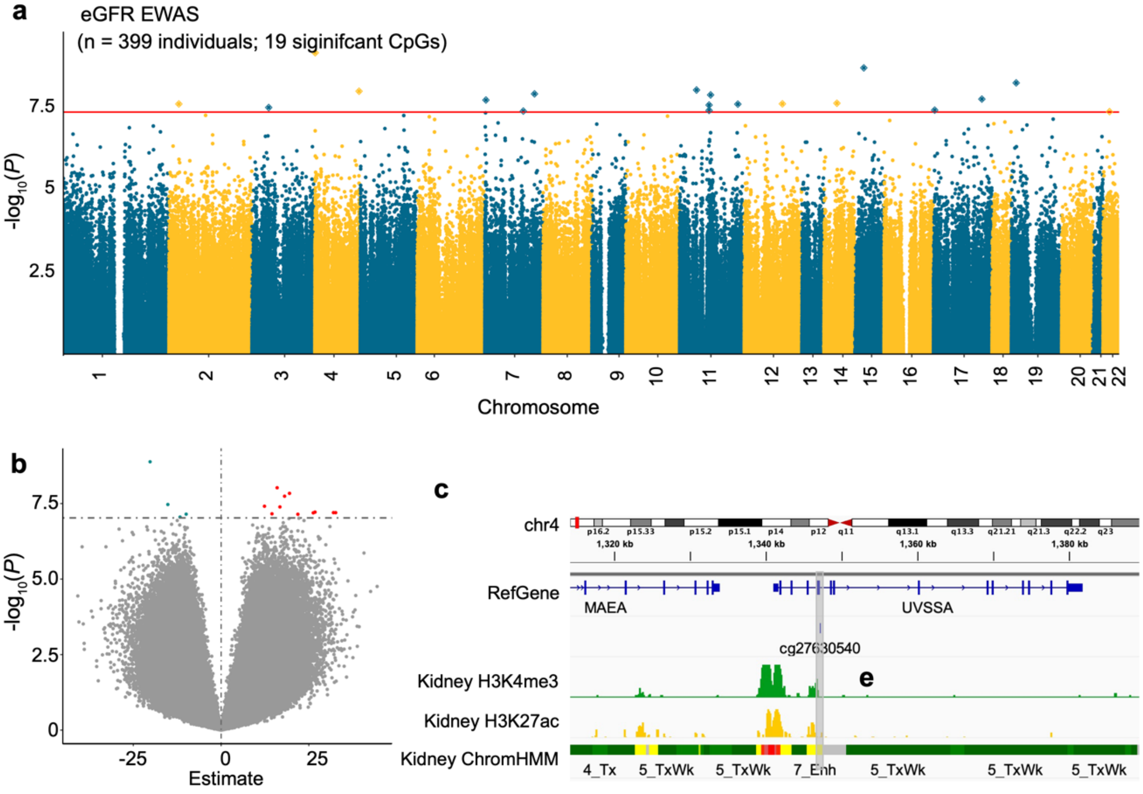

Supplemental Fig. 4: Directional consistency of the association between methylation and interstitial fibrosis.

a. The effect size for methylation and fibrosis correlation in EPIC data (x-axis) and in methylation fibrosis association effect size in validation cohort 1 (y-axis) (Gluck’s study). The effect size showed directional consistency at the 55 overlapped fibrosis-associated CpGs between the current study and Gluck's study.

b. The effect size for methylation and fibrosis correlation in EPIC data (x-axis) and in methylation fibrosis association effect size in validation cohort 1 (y-axis) (Ko’s study). The effect size showed directional consistency at the 85 overlapped fibrosis-associated CpGs between the current study and Ko's study.

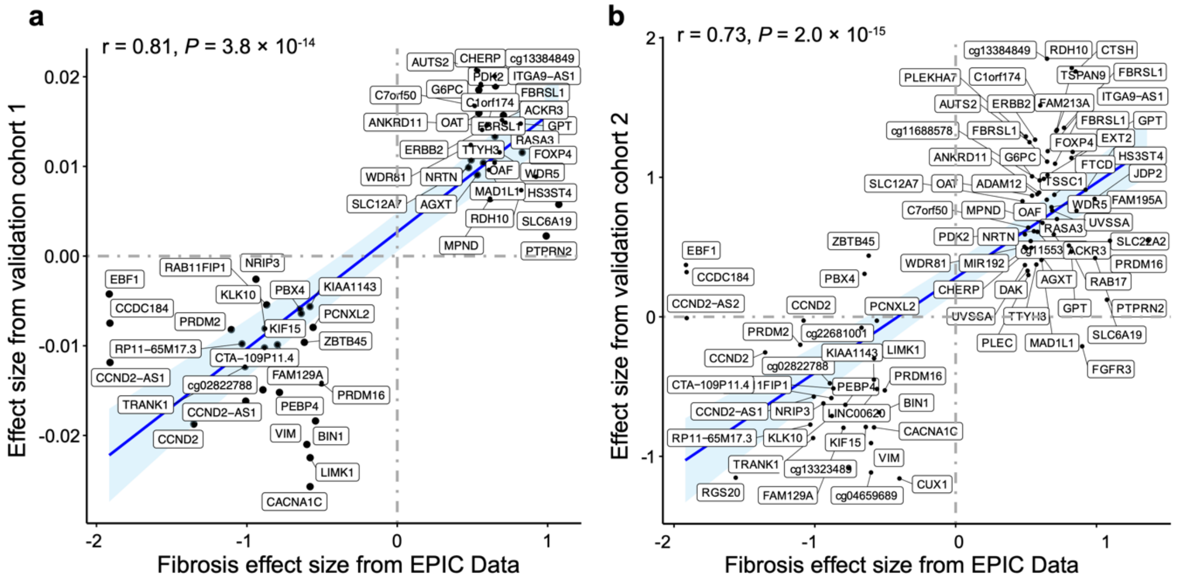

Supplemental Fig. 5: Sensitivity analysis of fibrosis and eGFR EWAS.

a. The Pearson’s correlation of effect sizes of the initial fibrosis EWAS model and the model that included BMI.

b. The Pearson’s correlation of effect sizes of the initial eGFR EWAS model and the model that included BMI.

c. The Pearson’s correlation of effect sizes of the initial fibrosis EWAS model and the model that included genetic PCs.

d. The Pearson’s correlation of effect sizes of the initial eGFR EWAS model and the model that included genetic PCs.

e. The Pearson’s correlation of effect sizes of the initial fibrosis EWAS and the analysis only included participants who had hypertension and/or diabetes.

f. The Pearson’s correlation of effect sizes of the initial eGFR EWAS and the analysis only included participants who had hypertension and/or diabetes.

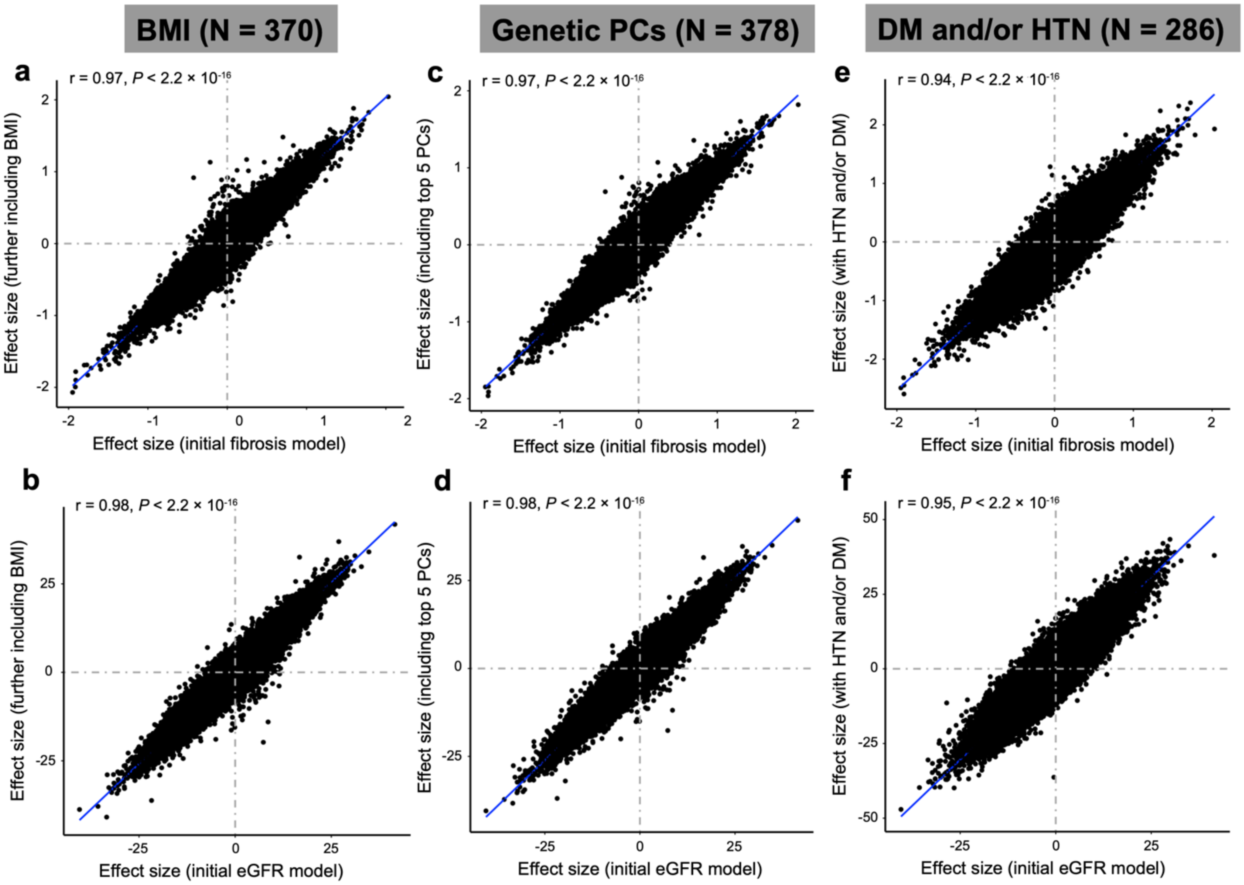

BMI (N = 370)
Genetic PCs (N = 378)
DM and/or HTN (N = 286)
a
r = 0.97, P < 2.2 × 10-16
Effect size (further including BMI)
Effect size (initial fibrosis model)
b
r = 0.98, P < 2.2 × 10-16
Effect size (further including BMI)
Effect size (initial eGFR model)
c
r = 0.97, P < 2.2 × 10-16
Effect size (including top 5 PCs)
Effect size (initial fibrosis model)
d
r = 0.98, P < 2.2 × 10-16
Effect size (including top 5 PCs)
Effect size (initial eGFR model)
e
r = 0.94, P < 2.2 × 10-16
Effect size (with HTN and/or DM)
Effect size (initial fibrosis model)
f
r = 0.95, P < 2.2 × 10-16
Effect size (with HTN and/or DM)
Effect size (initial eGFR model)

Supplemental Fig. 6: Human kidney snATAC-seq and snRNA-seq.

a. Gene activity score of cluster specific marker genes. The dot size represents the percentage of positive cells, and the color intensity indicates the average expression level. The data was replotted from Abedini et al.
b. Effect size and location of DAR in CKD. Control cell types were compared to CKD to identify cell-specific DARs. The significance was assessed using a 5% false discovery rate (FDR) q-value and an absolute log-fold-change threshold of 0.25.
c. Gene expression of cluster specific marker genes. The dot size represents the percentage of positive cells, and the color intensity indicates the average expression level. The data was replotted from Abedini et al.

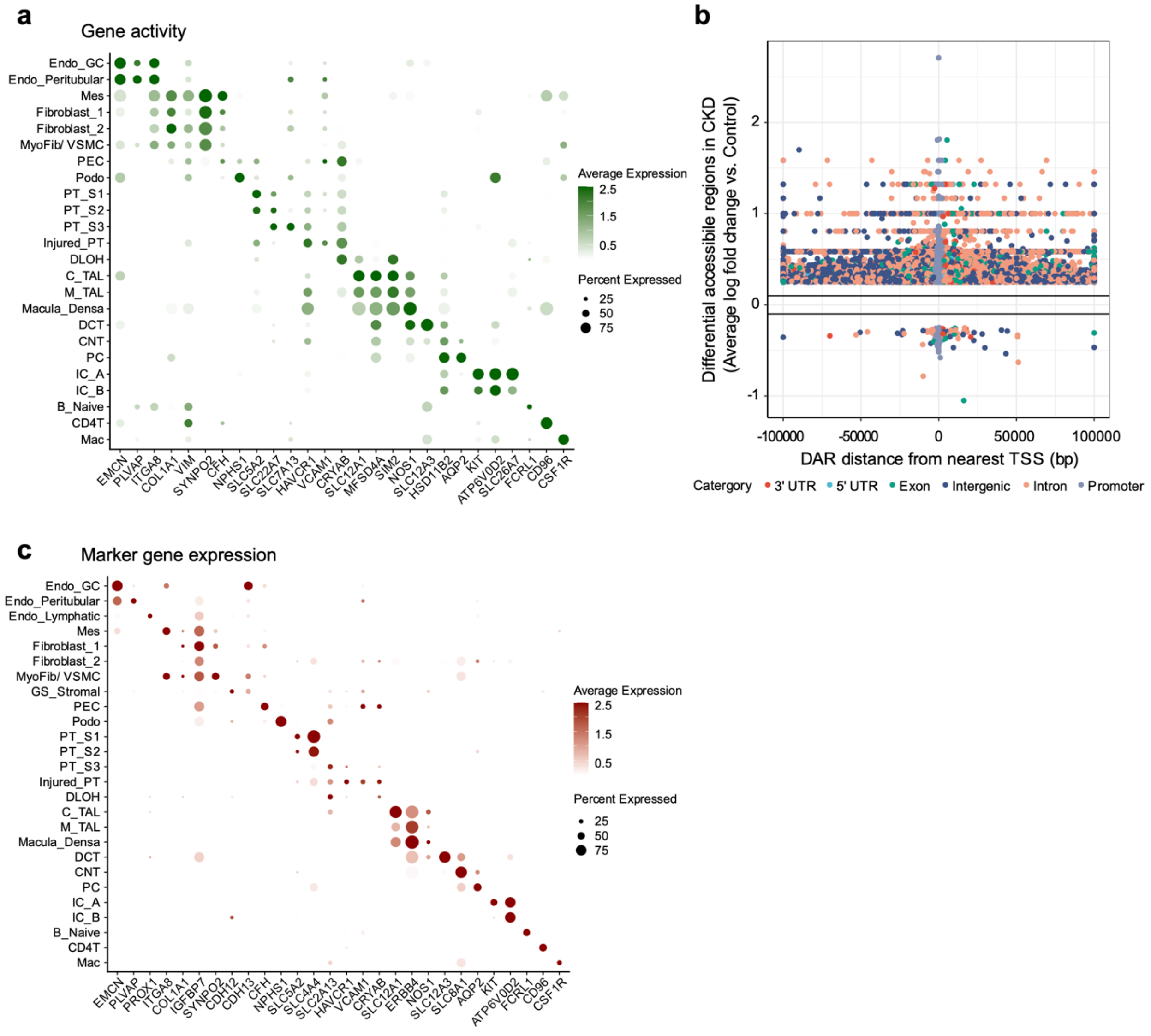

Supplementary Fig. 7. Functional enrichment of top 1000 fibrosis-associated DMPs' nearest genes.

a. Gene Ontology (GO) enrichment. The top 15 terms passing FDR < 0.05 are shown. The y-axis shows the enriched GO term ordered by enrichment *P* value (x-axis), and the color indicates the strength of enrichment (-log10(*P* value)) from strongest (red) to lowest (blue).

b. Reactome Pathway enrichment. The top 15 terms passing FDR < 0.05 are shown. The y-axis shows the enriched Reactome pathways ordered by enrichment *P* value (x-axis), and the color indicates the strength of enrichment (-log10(*P* value)) from strongest (red) to lowest (blue).

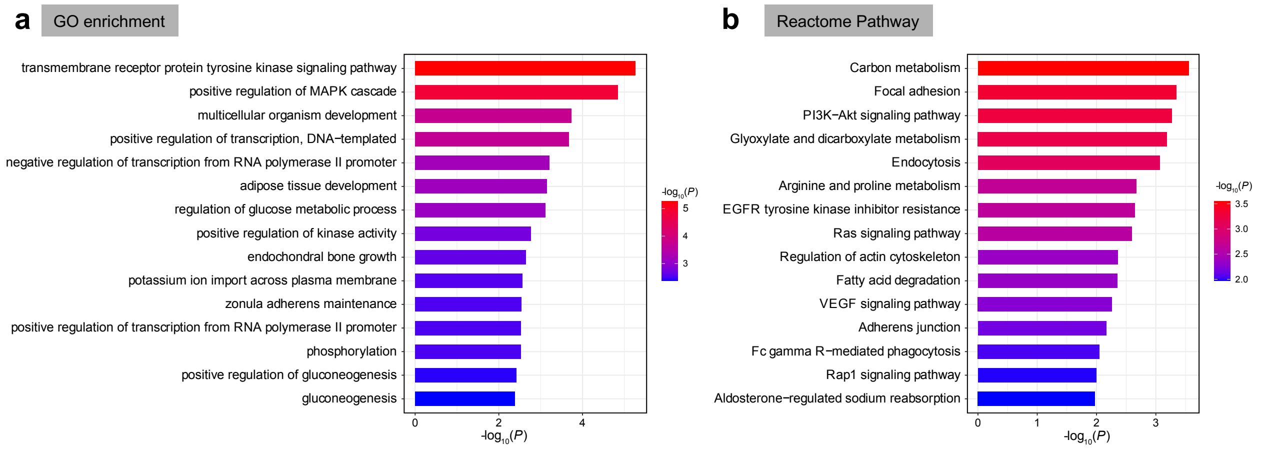

Supplementary Fig. 8. Epigenome-wide association analysis (EWAS) identified methylation changes associated with kidney function changes.

a. Manhattan plot of eGFR EWAS in 117 human kidney samples. The x-axis represents the chromosomal location of the CpG probes and the y-axis is the -log10(*P* value) of eGFR changes and methylation association. The epigenome-wide significance level ($P < 9.42 \times 10^{-8}$) is indicated by the red line and significant CpGs are highlighted as rectangles.

b. Volcano plot showing the association between eGFR changes and methylation changes. The x-axis represents the effect size (from the linear regression) of each CpG probe with eGFR changes and the y-axis indicates the strength of the association (-log10(*P* value)). Each dot corresponds to one probe, with red dots representing hypermethylated probes and cyan dots representing hypomethylated probes that are associated with higher eGFR changes.

c. Localization and characteristics of the most significant eGFR change EWAS signal at cg09514524. Top panel shows the chromosomal location and CCND2-AS1 gene followed by human kidney histone modification tracks (H3K4me3 and H3K27ac) followed by ChromHMM track.

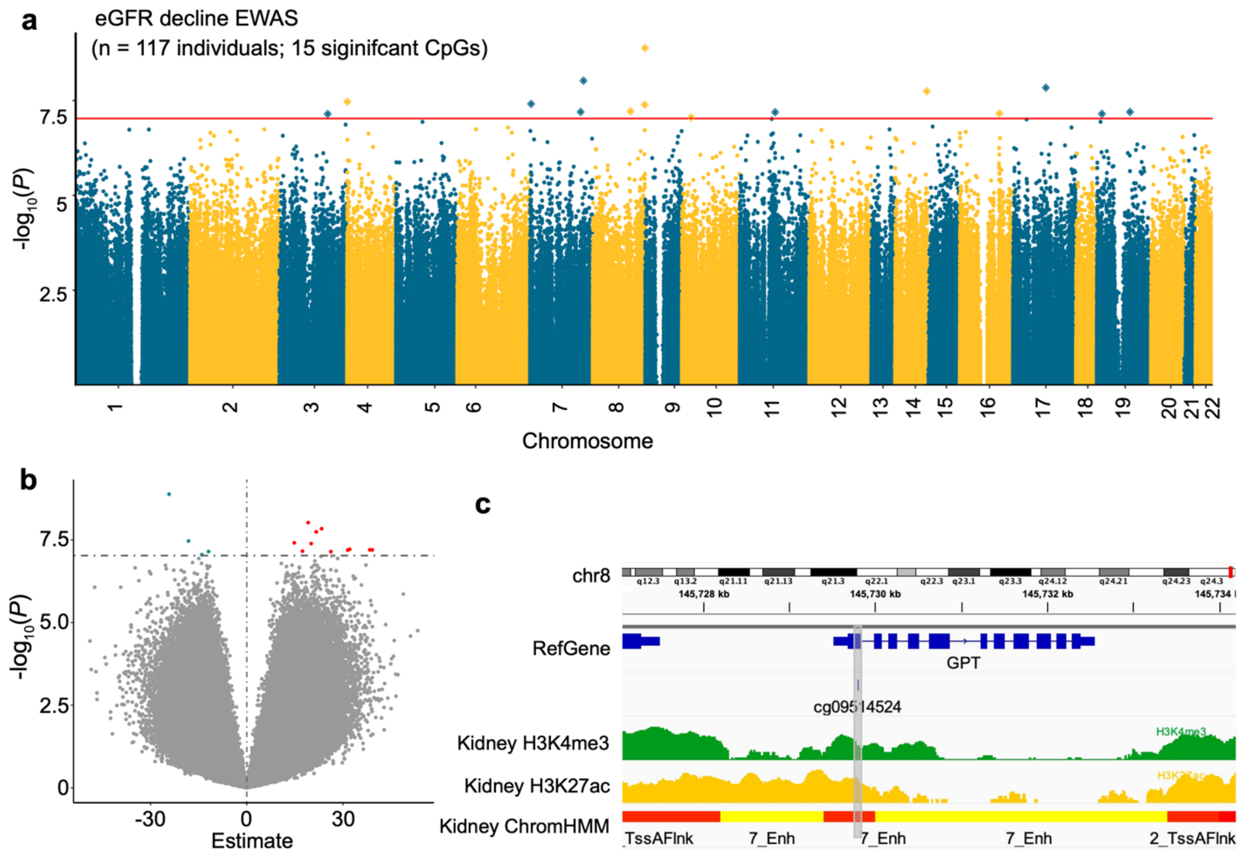

Supplement: Supplementary file 1 — Supplementary Information [file 41467_2024_45295_MOESM1_ESM.pdf]
