# Supplementary material for: Unraveling the epigenetic code: human kidney DNA methylation and chromatin dynamics in renal disease development
Source: Nat Commun. 2024 Jan 29;15:873. doi: 10.1038/s41467-024-45295-y (PMC10824731; doi:10.1038/s41467-024-45295-y)
Supplement: Supplementary file 3 — Description of Additional Supplementary Files [file 41467_2024_45295_MOESM3_ESM.pdf]

Description of Additional Supplementary Files

File Name: Supplementary Data 1
Description: A total of 171 CpG sites where cytosine methylation levels correlated with the degree of fibrosis at an epigenome-wide significance level (two-sided $P < 9.42 \times 10^{-8}$) after correcting for multiple testing and inflation.

File Name: Supplementary Data 2
Description: A total of 19 CpG sites where cytosine methylation levels correlated with eGFR at an epigenome-wide significance level (two-sided $P < 9.42 \times 10^{-8}$) after correcting for multiple testing and inflation.

File Name: Supplementary Data 3
Description: The demographic, clinical, and histopathological characteristics of the validation cohort 1.

File Name: Supplementary Data 4
Description: The demographic, clinical, and histopathological characteristics of the validation cohort 2.

File Name: Supplementary Data 5
Description: Comparison of the effect estimates and *P* values (two-sided) from epigenome-wide association analysis of the overlapping CpGs in our study and in the validation Cohort 1.

File Name: Supplementary Data 6
Description: Comparison of the effect estimates and *P* values (two-sided) from epigenome-wide association analysis of the overlapped CpGs in our study and in the validation Cohort 2.

File Name: Supplementary Data 7
Description: Enrichment of the 171 fibrosis significant differentially methylated CpGs in binding sites of transcription factors using HOMER. The P value was calculated by binomial test and was adjusted using the false discovery rate (FDR) method.

File Name: Supplementary Data 8
Description: A total of 3,825 SNPs around the 171 fibrosis-DMPs were identified as significant cis-meQTLs at FDR <0.01 level.

File Name: Supplementary Data 9
Description: The overlapping results of genetic variants that drove methylation differences (cis-meQTLs) with genetic variants associated with phenotype development (GWAS).

File Name: Supplementary Data 10
Description: Association between methylation-driving SNPs with nearby genes based on eQTL associations reported by Liu et al. (2022).

File Name: Supplementary Data 11
Description: Cell type specific accessible regions and gene activity in snATAC-seq.

File Name: Supplementary Data 12
Description: Differentially accessible chromatin regions between different cell types were identified using a likelihood ratio test, a log-fold-change threshold of 0.25, and an FDR of 0.05.

File Name: Supplementary Data 13
Description: Disease-associated cell-specific DARs were enriched for distinct transcription factors analyzed by the HOMER package.

File Name: Supplementary Data 14
Description: Transcription factors enrichment in genomic regions harboring fibrosis-associated DMPs in PT cells.

File Name: Supplementary Data 15
Description: Transcription factors enrichment (using HOMER) in Differentially accessible chromatin regions harboring fibrosis-associated DMPs in PT cells.

File Name: Supplementary Data 16
Description: Annotating fibrosis-associated DMPs to their nearest genes

File Name: Supplementary Data 17
Description: GO enrichment of fibrosis-associated DMPs annotated genes ($P<0.05$)

File Name: Supplementary Data 18
Description: Reactome pathway enrichment of fibrosis-associated DMPs annotated genes ($P<0.05$)

File Name: Supplementary Data 19
Description: Among the 171 fibrosis-DMPs, 13 were selected using the LASSO model. The penalty parameter λ was optimized using 10-fold cross-validation.

File Name: Supplementary Data 20
Description: Cytosine methylation levels at 15 CpG sites significantly correlated with changes in longitudinal eGFR at epigenome-wide significance ($P < 9.42 \times 10^{-8}$).
