# Supplementary material for: Unraveling the epigenetic code: human kidney DNA methylation and chromatin dynamics in renal disease development
Source: Nat Commun. 2024 Jan 29;15:873. doi: 10.1038/s41467-024-45295-y (PMC10824731; doi:10.1038/s41467-024-45295-y)
Supplement: Supplementary file 5 — Reporting Summary [file 41467_2024_45295_MOESM5_ESM.pdf]

nature portfolio

Corresponding author(s): Katalin Susztak
Last updated by author(s): Nov 20, 2023

Reporting Summary

Nature Portfolio wishes to improve the reproducibility of the work that we publish. This form provides structure for consistency and transparency in reporting. For further information on Nature Portfolio policies, see our Editorial Policies and the Editorial Policy Checklist.

Statistics

For all statistical analyses, confirm that the following items are present in the figure legend, table legend, main text, or Methods section.

n/a	Confirmed	
☐	☒	The exact sample size (*n*) for each experimental group/condition, given as a discrete number and unit of measurement
☐	☒	A statement on whether measurements were taken from distinct samples or whether the same sample was measured repeatedly
☐	☒	The statistical test(s) used AND whether they are one- or two-sided<br>*Only common tests should be described solely by name; describe more complex techniques in the Methods section.*
☐	☒	A description of all covariates tested
☐	☒	A description of any assumptions or corrections, such as tests of normality and adjustment for multiple comparisons
☐	☒	A full description of the statistical parameters including central tendency (e.g. means) or other basic estimates (e.g. regression coefficient) AND variation (e.g. standard deviation) or associated estimates of uncertainty (e.g. confidence intervals)
☐	☒	For null hypothesis testing, the test statistic (e.g. *F*, *t*, *r*) with confidence intervals, effect sizes, degrees of freedom and *P* value noted<br>*Give P values as exact values whenever suitable.*
☐	☒	For Bayesian analysis, information on the choice of priors and Markov chain Monte Carlo settings
☐	☒	For hierarchical and complex designs, identification of the appropriate level for tests and full reporting of outcomes
☐	☒	Estimates of effect sizes (e.g. Cohen's *d*, Pearson's *r*), indicating how they were calculated

Our web collection on statistics for biologists contains articles on many of the points above.

Software and code

Policy information about availability of computer code

Data collection	Image J software (v1.53) was used to take images of kidney tissue section stained with Hematoxylin and eosin and periodic acid schiff.
Data analysis	Kidney DNA methylation data preprocessing and quality control were performed using SeSAMe (v1.5.3). Linear mixed-effects models were employed to reduce potential batch effects that may influence DNA methylation measurements using the lmer function in the lme4 package (v 1.1-32, R version 4.2). EWAS analysis were performed using lm function (R version 4.2) with the linear models for DNA methylation data. BACON method was applied to reduce bias and inflation of the EWAS results using the bacon package (v 1.26.0, R version 4.2). Transcription factor motif enrichment was performed using HOMER (v4.10.3). snATAC-seq and snRNA-seq data: Cell Ranger ATAC (v. 1.1.0), Signac (v.1.3.0), Seurat (v. 4.3.0), and ChIPSeeker (v1.24.0). Methylation risk score was built used glmnet (v 4.1-7), ROCR (v 1.0-11), and lme4 (v 1.1.32).

For manuscripts utilizing custom algorithms or software that are central to the research but not yet described in published literature, software must be made available to editors and reviewers. We strongly encourage code deposition in a community repository (e.g. GitHub). See the Nature Portfolio guidelines for submitting code & software for further information.

Data

Policy information about availability of data

All manuscripts must include a data availability statement. This statement should provide the following information, where applicable:

- Accession codes, unique identifiers, or web links for publicly available datasets
- A description of any restrictions on data availability
- For clinical datasets or third party data, please ensure that the statement adheres to our policy

The human kidney snATAC-seq data have been deposited with the Gene Expression Omnibus (GEO) under accession code nos. GSE172008 (https://www.ncbi.nlm.nih.gov/geo/query/acc.cgi), GSE200547 (https://www.ncbi.nlm.nih.gov/geo/query/acc.cgi?acc=GSE200547) and the Common Metabolic Diseases Genome Atlas (https://cmdga.org/search/?type=Experiment&searchTerm=FNIH0000000). The Integrative Genomics Viewer visualization of human kidney cell-specific differentially accessible chromatin data generated in this study are provided at (https://susztaklab.com/Kidney_meQTL/index.php). Methylation data generated in this study have been deposited in Supplementary data 1 and Supplementary data 2. The raw individual participant data included in this project are protected and are not available due to data privacy laws. The human kidney eQTLs used in the present study are available online at the Susztaklab Kidney Biobank (https://susztaklab.com/Kidney_eQTL). The human kidney bulk RNA-seq data used in this study are available at GEO under accession numbers GSE115098 (https://www.ncbi.nlm.nih.gov/geo/query/acc.cgi?acc=GSE115098) and GSE173343 (https://www.ncbi.nlm.nih.gov/geo/query/acc.cgi?acc=GSE173343).

Research involving human participants, their data, or biological material

Policy information about studies with human participants or human data. See also policy information about sex, gender (identity/presentation), and sexual orientation and race, ethnicity and racism.

Reporting on sex and gender	Sex was self-reported and confirmed with the RNA-seq data. Sex was adjusted for the EWAS analysis, and reported whenever available.
Reporting on race, ethnicity, or other socially relevant groupings	Race was self-reported.
Population characteristics	This study included 399 kidney samples from healthy, diabetic, hypertensive, and diabetic and hypertensive CKD subjects. The mean age was 60.2 years. Approximately one-third of the participants had diabetes, and nearly 68% had hypertension. The mean eGFR, calculated by the CKD-EPI formula, was 71.2 ml/min/1.73m2, ranging from 3.7 to 134.9 ml/min/1.73m2. Approximately one-third (n=123) of the subjects had an eGFR less than 60 ml/min/1.72m2, meeting the classic definition of CKD. A mixed effect model was used to account for technical variation, encompassing bisulfite conversion control, mean intensity of measurements, sample plate, lymphocytic infiltration, and batch-specific variability. And a linear regression model adjusting for age, sex, race, diabetes, and hypertension was used to identified DMPs.
Recruitment	The primary cohort consisted of a cross-sectional evaluation of 506 human participants undergoing clinically indicated nephrectomies for renal neoplasia. Demographic, clinical information, and laboratory data were collected through an honest broker. After removing missing data, this study finally included 399 subjects with methylation measurements, kidney histological scores, and good-quality genotype data. Additionally, a subset of samples (117 subjects) had longitudinal kidney function measurements with at least 3 months of follow-up after nephrectomy.
Ethics oversight	Human kidney tissue collection was approved by the University of Pennsylvania Institutional Review Board, and no informed consent was obtained because the study was deemed IRB-exempt (exemption IV).

Note that full information on the approval of the study protocol must also be provided in the manuscript.

Field-specific reporting

Please select the one below that is the best fit for your research. If you are not sure, read the appropriate sections before making your selection.

☒ Life sciences ☐ Behavioural & social sciences ☐ Ecological, evolutionary & environmental sciences

For a reference copy of the document with all sections, see nature.com/documents/nr-reporting-summary-flat.pdf

Life sciences study design

All studies must disclose on these points even when the disclosure is negative.

Sample size	We conducted epigenome-wide analysis of 399 human kidney tissue samples, including controls, diabetic, hypertensive, and CKD tissues.
Data exclusions	Participants without kidney fibrosis score, eGFR, or methylation data were excluded.
Replication	In lack of a replication cohort with methylation data using EPIC array, we validated the fibrosis-DMPs in two independent external cohorts containing 91 and 85 human kidney samples obtained from individuals with and without diabetes and analyzed using Illumina Infinium 450K arrays.

Randomization	The study design was observational and therefor randomization was not relevant to the study.
Blinding	The study design was observational and therefor blinding was not relevant to the study.

Reporting for specific materials, systems and methods

We require information from authors about some types of materials, experimental systems and methods used in many studies. Here, indicate whether each material, system or method listed is relevant to your study. If you are not sure if a list item applies to your research, read the appropriate section before selecting a response.

Materials & experimental systems

n/a	Involved in the study
☒	☐ Antibodies
☒	☐ Eukaryotic cell lines
☒	☐ Palaeontology and archaeology
☒	☐ Animals and other organisms
☒	☐ Clinical data
☒	☐ Dual use research of concern
☒	☐ Plants

Methods

n/a	Involved in the study
☒	☐ ChIP-seq
☒	☐ Flow cytometry
☒	☐ MRI-based neuroimaging

Plants

Seed stocks	*Report on the source of all seed stocks or other plant material used. If applicable, state the seed stock centre and catalogue number. If plant specimens were collected from the field, describe the collection location, date and sampling procedures.*
Novel plant genotypes	*Describe the methods by which all novel plant genotypes were produced. This includes those generated by transgenic approaches, gene editing, chemical/radiation-based mutagenesis and hybridization. For transgenic lines, describe the transformation method, the number of independent lines analyzed and the generation upon which experiments were performed. For gene-edited lines, describe the editor used, the endogenous sequence targeted for editing, the targeting guide RNA sequence (if applicable) and how the editor was applied.*
Authentication	*Describe any authentication procedures for each seed stock used or novel genotype generated. Describe any experiments used to assess the effect of a mutation and, where applicable, how potential secondary effects (e.g. second site T-DNA insertions, mosiacism, off-target gene editing) were examined.*
